# Is the EMpressin Injection in ENDOmetrioma eXcision Surgery Useful? The EMENDOX Study

**DOI:** 10.3390/jcm14217716

**Published:** 2025-10-30

**Authors:** Flavia Pagano, Ioannis Dedes, Cloé Vaineau, Franziska Siegenthaler, Sara Imboden, Michael David Mueller

**Affiliations:** Department of Obstetrics and Gynecology, University Hospital of Berne, University of Bern, 3012 Bern, Switzerland; ioannis.dedes@ksgl.ch (I.D.); cloe.vaineau@insel.ch (C.V.); franziska.siegenthaler@insel.ch (F.S.); saraimboden@gmx.ch (S.I.); michel.mueller@insel.ch (M.D.M.)

**Keywords:** endometrioma, cystectomy, Empressin, recurrence rate, hormonal therapy

## Abstract

**Background**: Endometrioma recurrence after laparoscopic cystectomy remains a clinical challenge in the long-term management of endometriosis. The Empressin Injection Technique (EIT), which involves the use of a vasopressin analog during surgery, may reduce recurrence by improving the completeness of cyst removal. This study aimed to evaluate the impact of the EIT on recurrence rates compared to standard cystectomy without Empressin. **Methods**: We conducted a retrospective case–control study of 263 patients who underwent laparoscopic cystectomy for unilateral or bilateral endometriomas between 2014 and 2024 at a tertiary endometriosis referral center. The patients were divided into two groups: EIT (n = 110) and control (n = 153). In the EIT group, 10 mL of diluted Empressin (1 mL in 100 mL NaCl 0.9%) was injected at the cyst capsule–ovarian cortex interface prior to stripping. Demographic and clinical variables were analyzed using descriptive statistics (chi-square test and the Mann–Whitney *t*-test) and logistic regression to identify factors associated with recurrence between the two groups. **Results**: No significant differences were found between the groups regarding age, BMI, #ENZIAN O score, or r-ASRM stage. No intraoperative or postoperative complications were reported. Recurrence was significantly lower in the EIT group (5.5%) compared to the control group (19.6%) (*p* = 0.001; OR 0.2, 95% CI: 0.08–0.55). Hormonal therapy was administered postoperatively in 69.1% of EIT patients and 62.5% of controls. Pregnancy rates were comparable between the groups. Repeat surgery for recurrence was required only in the control group (4.2%, *p* = 0.004). **Conclusions**: Use of Empressin during laparoscopic cystectomy significantly reduces endometrioma recurrence without adverse effects, particularly when combined with postoperative hormonal therapy.

## 1. Introduction

Endometriosis is a common gynecological condition characterized by the presence of endometrium-like glands and stroma outside the uterine cavity. It affects approximately 6% to 10% of women of reproductive age [1,2]. Endometriosis can manifest in several forms, including peritoneal implants, adhesions, deeply infiltrating lesions, and ovarian endometriomas (OMAs) [3]. Ovarian endometriomas occur in 17–44% of women with endometriosis, significantly affecting quality of life, leading to chronic pelvic pain and infertility, and increasing the risk of malignancy [4,5].

The pathogenesis of ovarian endometriomas (OMAs) remains debated. In 1921, Sampson proposed that OMAs develop when endometrial tissue invades ovarian cysts through retrograde menstruation and subsequent implantation [6], while later theories suggested metaplasia of the ovarian surface epithelium or invagination of superficial implants into the ovarian cortex [4,7,8]. The management of endometriomas aims to relieve symptoms, exclude malignancy, prevent ovarian torsion, preserve ovarian endocrine function, minimize the risk of recurrence, and optimize fertility outcomes [4]. Observation may be appropriate for asymptomatic patients considered at low risk for torsion or malignancy, based on factors such as cyst size (<5 cm), benign imaging features, patient age, and family history [4].

Current international guidelines (ESHRE and NICE) do not recommend routine preoperative hormonal therapy for ovarian endometriomas but instead support an individualized approach [9]. At our institution, we apply a tailored management protocol that includes short-term hormonal therapy—typically dienogest for four to eight weeks prior to surgery—to reduce cyst size and inflammation, thereby optimizing surgical conditions [10]. Serum Anti-Müllerian Hormone (AMH) levels are routinely assessed to evaluate ovarian reserve and to provide individualized fertility counseling, including oocyte cryopreservation for women with low AMH levels. In asymptomatic patients, dienogest may also be prescribed as a preventive measure to limit disease progression and preserve ovarian reserve over time.

Surgical options for the treatment of ovarian endometriomas include cystectomy, drainage with ablation, or sclerotherapy [11,12], among which cystectomy has been shown to significantly reduce the recurrence rate, reoperation, and symptoms such as dysmenorrhea, dyspareunia, and chronic pelvic pain [13].

However, during laparoscopic cystectomy, stripping and coagulation may cause thermal injury to ovarian follicles, thereby reducing the ovarian reserve [14,15]. The risk of such injury is higher in the case of small (<3 cm) fibrotic cysts, for which the dissection plane is unclear, and in large (>5 cm) OMAs that adhere to adjacent structures [4]. Despite the transient postoperative decline in AMH levels, cystectomy remains associated with higher spontaneous pregnancy rates and lower recurrence compared with ablation or sclerotherapy [16]. 

Several studies have investigated the efficacy and feasibility of “vasopressin injection-assisted laparoscopic excision of ovarian endometriomas” [11]. The injection of diluted Empressin into the interface between the cyst wall and the ovarian cortex facilitates dissection by creating a clearer cleavage plane and inducing local vasoconstriction. This approach reduces intraoperative bleeding, limits the need for coagulation, and consequently minimizes thermal injury and inadvertent removal of healthy ovarian tissue, thereby helping to preserve the postoperative ovarian reserve [14,17].

Overall recurrence rates after conservative surgery for ovarian endometriomas increase over time, reaching approximately 20% at two years and up to 40–50% at five years, according to observational series and systematic reviews [18,19,20,21]. In contrast, after excisional surgery (cystectomy), reported one-year recurrence rates typically range between 5% and 17%, which are lower than those observed with ablative techniques, for which the one-year recurrence rate may reach 37%. These rates vary depending on the surgical approach, definition of recurrence, and length of follow-up [22]. Reported risk factors for recurrence include younger age, bilateral cysts, large cyst size, prior endometriosis surgery, and the use of conservative techniques, such as ablation or sclerotherapy [18,23,24]. Recurrence may result from the regrowth of residual lesions or de novo implantation via retrograde menstruation [25]. Postoperative hormonal therapy can suppress residual microscopic disease and further reduce the risk of recurrence [4,9,21,26,27].

## 2. Materials and Methods

This retrospective case–control study included women who underwent surgery for endometriosis at the certified endometriosis center of Bern University Hospital between January 2014 and April 2024. The inclusion criteria were premenopausal women who underwent laparoscopic cystectomy for unilateral or bilateral ovarian endometrioma using either the Empressin Injection Technique (EIT) or standard cystectomy, with the surgical intent of complete excision of all visible endometriotic lesions. Histopathological confirmation of endometrioma and a minimum postoperative follow-up period of ≥6 months were required. The exclusion criteria were postmenopausal status, histological findings other than ovarian endometrioma, use of alternative surgical techniques (e.g., laser ablation, drainage/ablation, or sclerotherapy), concomitant malignant ovarian pathology, incomplete excision or staged procedures, and insufficient follow-up (<6 months) or missing outcome data.

We aimed to compare the OMA recurrence rate in women who underwent laparoscopic cystectomy with or without Empressin injection. Demographic data, prior surgical and medical history, and preoperative symptoms were collected for all patients. Ultrasound scans were also performed, and a preoperative MRI was conducted if deep infiltrating endometriosis was suspected. The following variables were analyzed: patient demographics, age, preoperative symptoms, surgical procedures, endometriosis stage, fertility status, location and distribution of endometriotic lesions, and postoperative complications. All patients were followed for at least six months postoperatively.

**Figure 1:**
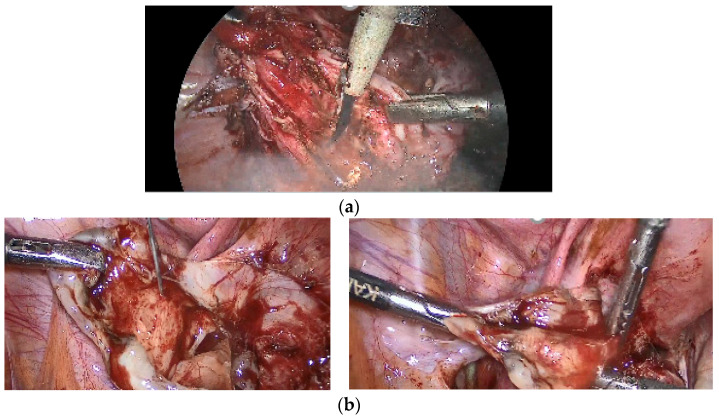
(**a**) Simple cystectomy; (**b**) cystectomy with Empressin.

In both groups, we included patients who underwent complete surgical excision of endometriosis with the aim of removing all endometriotic lesions. The patients were divided into two groups: an EIT (Empressin Injection Technique) group and a control group (standard cystectomy without the EIT). All surgeries were performed under general anesthesia by experienced endometriosis surgeons, using a standardized surgical protocol to ensure consistency and minimize inter-operator variability. In the EIT group, 10 mL of a diluted Empressin solution (1 mL Empressin diluted in 100 mL Nacl 0.9%) was injected into the interface between the cyst capsule and the ovarian cortex after aspirating the endometrioma’s fluid and before stripping and coagulation. In the control group, no Empressin injection was administered. The cyst capsule was directly exposed using scissors, and once the cleavage plane was identified, two grasping forceps were used to carefully separate the normal ovarian tissue from the cyst wall until complete removal. Bipolar forceps were used for hemostasis as required, and ovarian suturing was not performed. Figure 1 shows the surgical technique.

All other aspects of the surgical procedure were consistent across both groups.

A retrospective design was chosen because the Empressin Injection Technique (EIT) was routinely used in our surgical practice during the study period. This approach allowed us to evaluate a large, homogeneous cohort of patients treated under real-world conditions, assessing the feasibility, safety, and clinical outcomes of the technique before planning prospective or randomized studies.

### Statistical Analysis

Statistical analyses were performed using SPSS version 25.0 (IBM^®^, Armonk, NY, USA). Demographic and clinic-pathologic characteristics were compared between groups using descriptive statistics. Categorical variables were analyzed using the chi-square test, and continuous variables were analyzed using the Mann–Whitney U test. A two-sided *p* value < 0.05 was considered statistically significant. Logistic regression analysis was performed to evaluate the relationship between patient characteristics and the recurrence of ovarian endometrioma (expressed as an odds ratio). Postoperative outcomes were analyzed using the Kaplan–Meier method with Cox regression to estimate hazard ratios.

## 3. Results

### 3.1. Patient Characteristics

During the study period (2014–2024), 2093 patients underwent surgery for endometriosis, of whom 263 underwent laparoscopic cystectomy for unilateral or bilateral OMAs and were followed at our center. Demographic and surgical data are summarized in Table 1.

**Figure 2:**
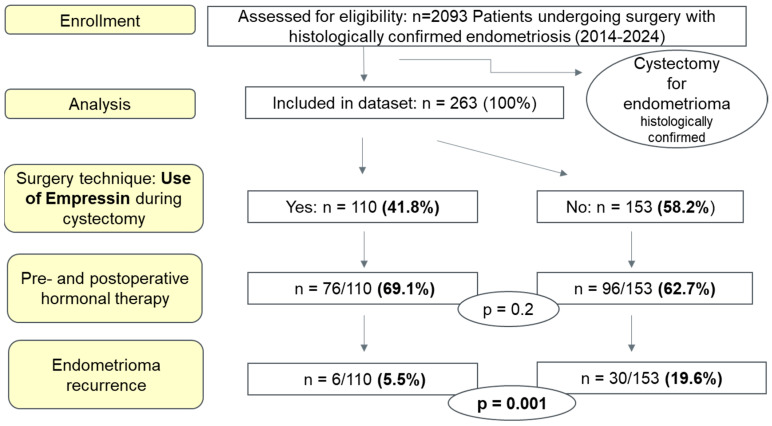
Study flowchart: design, recurrence rates, and hormonal therapy timing.

Figure 2 presents a flowchart of patient selection, postoperative recurrence, and the proportion of patients receiving hormonal therapy before and after surgery.

Endometriosis severity was classified according to both the revised American Society for Reproductive Medicine (r-ASRM) classification and the #ENZIAN classification system.

The r-ASRM stage (I–IV) provides an overall assessment of disease extent, based on lesion size, location, and the presence of adhesions involving the ovaries, fallopian tubes, and peritoneum.

**Figure 3:**
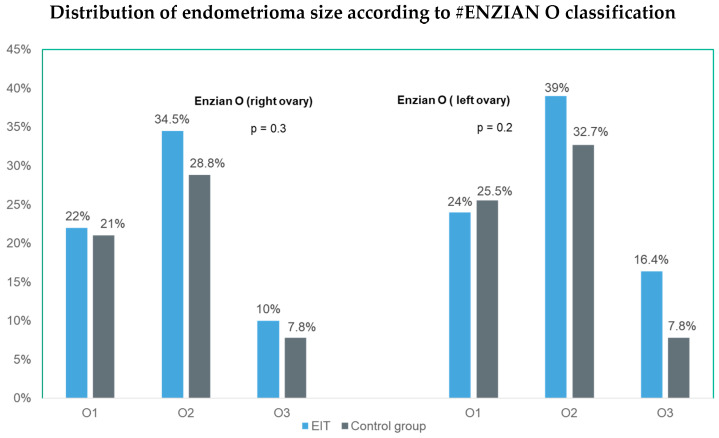
Comparison of endometrioma size between the EIT and control groups according to the #ENZIAN O classification. The chart shows the percentage of patients in each O category: O1 (<3 cm), O2 (3–7 cm), and O3 (7 cm).

The #ENZIAN classification complements the r-ASRM assessment by providing a detailed description of deep and ovarian endometriosis. Specifically, the O compartment (O score) of the ENZIAN system quantifies the size of ovarian endometriomas as follows: ENZIAN O1—lesions < 3 cm in diameter; ENZIAN O2—lesions between 3 and 7 cm; ENZIAN O3—lesions > 7 cm. In our study, cyst sizes were compared between the EIT and control groups according to the ENZIAN O classification to ensure comparability of lesion size. No significant differences were found in age, BMI, r-ASRM stage (Table 1), or mean endometrioma diameter between the two groups based on the #ENZIAN O score (Figure 3).

### 3.2. Surgical Procedure and Operative Time

The mean duration of Empressin injection in the EIT group was 1.11 min. The total operative time, including the injection phase, averaged 14.95 min in the EIT group. When excluding the injection phase, the mean cystectomy duration was 13.84 min in the EIT group and 12.44 min in the control group.

Statistical analysis revealed no significant difference in total operative time between the EIT and control groups (t = 1.18, *p* = 0.2480), nor in cystectomy duration when the injection time was excluded (t = 0.66, *p* = 0.5151). These findings suggest that the use of Empressin did not significantly prolong the surgical procedure [28]. The slightly longer cystectomy time observed in the EIT group may reflect the additional technical step of injection and the potential impact of vascular modulation on tissue dissection. Overall, the data support the feasibility of using Empressin without compromising surgical efficiency.

### 3.3. Surgical and Postoperative Outcomes

All intraoperative and postoperative complications were prospectively documented. No ovary-related surgical complications were observed in this cohort. Specifically, no oophorectomies were required, and no cases of postoperative ovarian failure occurred. Premature Ovarian Insufficiency (POI) is defined as the loss of normal ovarian function before the age of 40, characterized by amenorrhea or oligomenorrhea for at least four months and elevated gonadotropin levels (FSH > 25 IU/L on two separate occasions ≥ four weeks apart), with or without low estradiol levels. POI may present alongside symptoms of estrogen deficiency, such as hot flashes, vaginal dryness, and infertility. None of the patients reported amenorrhea, oligomenorrhea, or menopausal symptoms. In case of suspected menstrual irregularity, hormonal profiles (FSH, LH, and estradiol) were evaluated and found to be within the normal premenopausal range. Among patients without an immediate desire for pregnancy, postoperative hormonal therapy was prescribed for secondary prevention of endometriosis-related symptoms and recurrence. Specifically, 69.1% (76/110) of patients in the Empressin group and 62.5% (96/153) in the control group received postoperative hormonal treatment (Figure 2).

All patients underwent ultrasound follow-up after surgery. Recurrence of ovarian endometrioma was defined as the reappearance of a round, thick-walled cyst within the ovary, containing low-echogenic fluid and measuring ≥1.0 cm in diameter. Recurrence was observed in 5.5% (6/110) of patients in the Empressin group compared with 19.6% (30/153) in the control group, representing a statistically significant difference (*p* = 0.001, OR 0.2, 95% CI: 0.08–0.55). The mean time to last follow-up or recurrence was 53.6 months in the Empressin group and 66.6 months in the control group (*p* > 0.05). No significant difference in pregnancy rate was found between the two groups (34.4%, 22/110 in the EIT group, 23.6%, 21/153 in the control group, *p* > 0.05). A second surgery for recurrence was required in 4.2% (11/153) of patients in the control group only (*p* = 0.004). Although recurrence tended to increase with cyst size (based on the #ENZIAN O score), this association was not statistically significant (OR 1.20, 95% CI 0.84–1.75, *p* = 0.29).

In the EIT group, bilateral endometriomas were present in 45.5% (50/110) of patients, compared with 24.2% (37/153) in the control group (*p* < 0.001). Among the patients with bilateral endometriomas, recurrence occurred in 4% (2/50) of the EIT group versus 24.3% (9/37) of the control group (*p* < 0.02).

A multivariate logistic regression analysis was performed to evaluate the risk of recurrence between the two groups, considering Empressin use as the main independent variable, adjusted for age, BMI, cyst size (according to the Enzian score), and hormonal therapy (pre- and postoperative). Our analysis revealed that Empressin use was significantly associated with a lower recurrence rate (*p* = 0.001, OR 0.2, 95% CI 0.07–0.52). Postoperative hormonal therapy showed a trend toward significance (*p* = 0.061). Even after adjusting for age, BMI, cyst size, and hormonal therapy, Empressin use remained significantly associated with a lower recurrence risk (Table 2).

**Figure 4:**
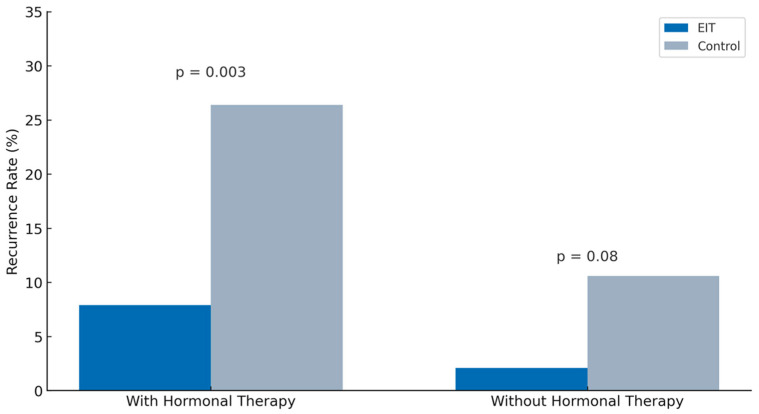
Comparison of recurrence rates between EIT and control groups.

Recurrence rates were further analyzed according to the use of postoperative hormonal therapy (Figure 4). Among the 110 patients in the EIT group, 63 (57.3%) received postoperative hormonal therapy, compared with 87 of 153 patients (56.9%) in the control group. In this subgroup, 5 patients (7.9%) in the EIT group and 23 patients (26.4%) in the control group experienced recurrence, a statistically significant difference (*p* = 0.003).

Among the patients who did not receive postoperative hormonal therapy, 47 of 110 (42.7%) in the EIT group and 66 of 153 (43.1%) in the control group received no treatment. In this subgroup, recurrence was observed in one patient (2.1%) in the EIT group and in seven patients (10.6%) in the control group; this difference did not reach statistical significance (*p* = 0.08).

These findings suggest that the use of the Empressin Injection Technique (EIT) is associated with lower recurrence rates, particularly when combined with postoperative hormonal therapy.

Table 3 shows the recurrence rate in the two groups at 6, 12, 24, 36, and 48 months.

**Figure 5:**
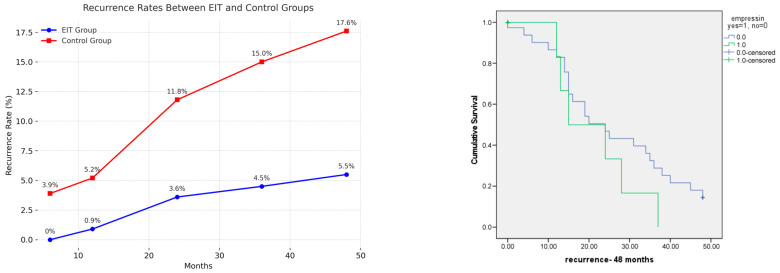
Kaplan–Meier survival plot of 48-month recurrence-free survival for EIT and control groups.

All recurrences in the EIT group and the majority of those in the control group (27 of 30) occurred within 48 months of surgery (Figure 5).

**Figure 6:**
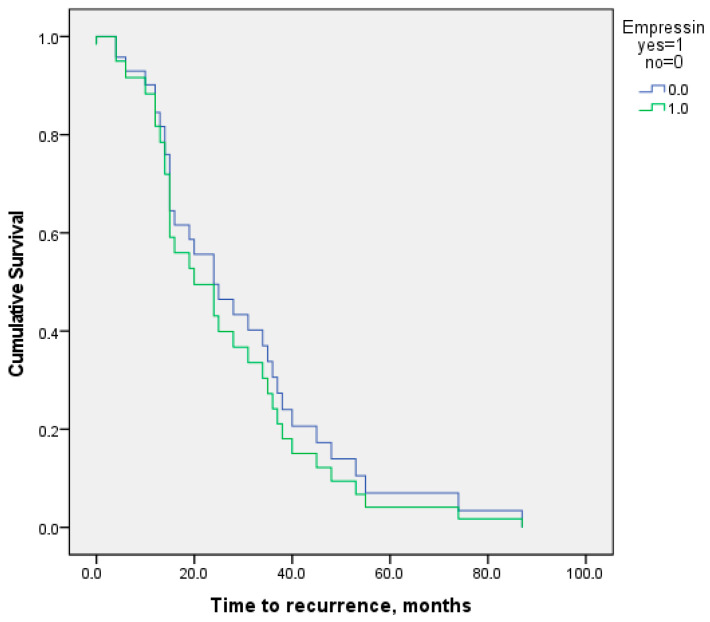
Kaplan–Meier curve for time to recurrence in patients undergoing cystectomy with or without Empressin.

Finally, a Cox regression analysis was performed to assess whether potential confounding factors—such as age at surgery, BMI, follow-up duration, and the distribution of endometriotic lesions according to the ENZIAN score—had an impact on recurrence risk between groups. None of these variables showed a statistically significant association with recurrence (all *p* > 0.05), suggesting that these factors did not influence the observed relationship between Empressin use and recurrence risk (Figure 6).

## 4. Discussion

This study aimed to evaluate the effect of the Empressin Injection Technique (EIT) on the rate of OMA recurrence following laparoscopic cystectomy. Our findings indicate that the use of Empressin significantly reduced the OMA recurrence rate compared with conventional laparoscopic cystectomy without Empressin, with a recurrence rate of 5.5% in the Empressin group versus 19.6% in the control group (*p* = 0.001). This statistically significant difference suggests that Empressin injection may play a key role in reducing the postoperative recurrence of OMAs.

### 4.1. Impact of Empressin on Recurrence Rate

By creating a clearer dissection plane between the cyst wall and the ovarian cortex through hydrodissection and local vasoconstriction, Empressin reduces bleeding, limits the need for coagulation, and minimizes thermal damage to healthy ovarian tissue. This mechanism likely decreases the risk of residual endometriotic tissue, thereby lowering recurrence rates. Moreover, by facilitating precise dissection, Empressin helps to preserve normal ovarian parenchyma, particularly in cases of fibrotic or multilocular cysts in which the cleavage plane is poorly defined [29,30].

Previous studies reported similar findings. Saeki et al. (2010) showed that the injection of diluted vasopressin into the interface between the ovarian capsule and cyst wall reduced intraoperative bleeding and the need for coagulation during cystectomy [29]. Although its impact on ovarian reserve remains debated [17], in our study, no intraoperative or postoperative complications related to the use of Empressin were observed. Additionally, there were no reports of premature ovarian failure during the 12-month follow-up for all patients. The mean time to the last follow-up or recurrence was 53.6 months in the Empressin group and 66.6 months in the control group (*p* > 0.05). Furthermore, there was no significant difference in pregnancy rates between the two groups.

### 4.2. Postoperative Hormonal Therapy and Its Synergistic Effect with Empressin

Consistent with the existing literature, excisional surgery (cystectomy) is associated with a lower one-year recurrence rate (5–17%) compared with ablative techniques (up to 37%), although recurrence increases cumulatively with longer durations of follow-up [11,22]. According to the recent systematic review and meta-analysis by Veth et al. (2024), the rate of OMA recurrence after surgery without postoperative hormonal therapy reached a weighted mean of 27% at 24 months, supporting the well-established role of hormonal suppression in reducing recurrence [31,32]. In our study, recurrence rates were significantly lower among patients who received postoperative hormonal therapy. Specifically, recurrence occurred in 5 of 63 patients (7.9%) in the EIT group compared with 23 of 87 (26.4%) in the control group (*p* = 0.003), despite similar rates of hormonal therapy use in both groups. This suggests that Empressin may offer additional benefits in reducing recurrence when combined with hormonal therapy.

Interestingly, even among patients who did not receive hormonal therapy, the recurrence rate remained lower in the EIT group (2.1%) than in the control group (10.6%), although this difference did not reach statistical significance (*p* = 0.08). These findings imply that the surgical technique itself—particularly the use of Empressin—may independently contribute to reducing the risk of recurrence, albeit with a less pronounced effect in the absence of postoperative hormonal suppression.

The combination of intraoperative Empressin and postoperative hormonal therapy may have a synergistic effect: Empressin facilitates more complete cyst wall excision through vasoconstriction and hydrodissection, while hormonal therapy suppresses residual disease by creating a hypoestrogenic environment. Together, these mechanisms may provide more effective long-term control of recurrence than either intervention alone. Previous studies highlighted the superiority of GnRH agonists and progestins over oral contraceptives in preventing recurrence [3,21]. In our cohort, 150 of 263 patients (57%) received postoperative hormonal therapy, most commonly dienogest (82%, 123/150), followed by combined oral contraceptives (2.7%, 4/150) and GnRH analogs (2%, 3/150). Among our patients, dienogest was the most well-tolerated medication. The choice of hormonal therapy should be individualized according to the patient’s profile, side effects, and treatment goals. Adherence to hormonal treatment remains crucial, as women with ovarian endometriomas are often undertreated hormonally compared to those with other endometriosis phenotypes [33]. In our study, adherence rates were high, with postoperative hormonal therapy used by 69.1% of patients in the EIT group and 62.7% in the control group.

### 4.3. Cystectomy Versus Laser Ablation

According to a Cochrane review by Kalra et al. (2024), cystectomy is more effective than ablative techniques, with lower recurrence rates (5–17% vs. 37% at 12 months), better pain relief, and higher pregnancy rates [22]. Nevertheless, concerns remain regarding the potential decline in ovarian reserve after excisional surgery. Our findings suggest that Empressin does not negatively affect fertility potential after laparoscopic cystectomy. On the contrary, by facilitating more precise dissection and minimizing thermal injury to healthy ovarian tissue, the Empressin technique may optimize the balance between surgical radicality and ovarian preservation.

### 4.4. Expanding the Role of Empressin in Gynecological Surgery

Based on our clinical experience, diluted Empressin may also be useful in adhesiolysis, facilitating the identification of tissue planes and safer dissection. Similarly, its application could be explored in the management of deep infiltrating endometriosis (DIE) nodules, where clear delineation of tissue layers is essential for complete excision while preserving adjacent structures. In addition, vasopressin has long been used in myomectomy to minimize intraoperative blood loss [34], and a recent randomized controlled pilot trial supports the safety and efficacy of diluted vasopressin in this context [35]. Overall, the Empressin^®^ technique appears to be a versatile tool that may provide hemostatic and technical advantages not only in ovarian cystectomy but also in adhesiolysis, deep endometriosis surgery, and myomectomy.

### 4.5. Cost–Benefit Considerations

Although the Empressin Injection Technique (EIT) introduces a minor additional material cost, its economic impact appears negligible in the context of the overall surgical procedure. In our study, 10 mL of diluted Empressin solution (prepared by diluting 1 mL of Empressin in 100 mL of 0.9% NaCl) was used per procedure, corresponding to an effective dose of approximately 0.1 mL of Empressin and an estimated cost of CHF 25 per intervention at our institution. Given the significant reduction in the recurrence rate observed with the EIT and the absence of adverse events or prolonged operative time, this modest expense may be considered highly cost-effective. Furthermore, the potential for improved surgical outcomes and reduced need for repeat surgery could enhance its cost–benefit profile. Future prospective studies including a formal cost-effectiveness analysis are warranted to confirm these findings.

### 4.6. Contraindications and Safety Considerations

Although there are no absolute contraindications to local vasopressin (Empressin^®^) injection, caution should be exercised in patients with cardiovascular comorbidities. Rare but severe systemic reactions—such as bradycardia, vasospasm, pulmonary edema, and cardiac arrest—have been reported, mainly due to intravascular absorption or excessive dosing [36,37]. Its use should therefore be avoided in patients with unstable ischemic heart disease, uncontrolled hypertension, severe vascular disease, or major conduction disorders, and the EIT should always be performed with a standardized diluted solution, careful aspiration, slow injection, and continuous intraoperative monitoring [37].

### 4.7. Learning Curve and Operator Requirements

The preparation and injection of diluted vasopressin are technically simple and can be learned quickly by surgeons familiar with laparoscopy, with evidence of reduced bleeding during cystectomy or myomectomy [17,38]. Nevertheless, its safe use in complex procedures requires advanced laparoscopic skills, particularly for correct tissue plane identification and the prevention of intravascular injection. Experienced surgeons can adopt the technique after a short familiarization period, whereas less experienced operators should initially perform it under supervision, consistent with the general principle that outcomes in advanced laparoscopic surgery improve with experience.

### 4.8. Strengths and Limitations

To our knowledge, this is the first study to investigate the efficacy of Empressin use during laparoscopic cystectomy for OMAs and its effect on recurrence with a long follow-up period. The strengths of this study include its large sample size, uniform surgical technique, and comprehensive pre- and postoperative data. Both groups underwent identical surgical procedures aside from the Empressin injection, ensuring that differences in outcome were more likely due to the Empressin intervention rather than variations in surgical technique. The use of robust statistical methods, such as logistic regression and Cox regression analysis, provides confidence in the reliability of the results. The limitations of this study include its retrospective nature and non-randomized design, which may have introduced bias. The non-randomized design also meant that confounding factors, such as patient characteristics, could not be fully controlled. Additionally, a follow-up disparity between the EIT (mean follow-up of 53.6 months) and control (66.6 months) groups could result in underestimation of late recurrences in the EIT group >5 years. The evidence strongly supports that Empressin reduces OMA recurrence independent of follow-up duration, but future studies with matched follow-up times are required to strengthen this conclusion.

Notably, our study has the longest follow-up presented in the literature to date, and we demonstrate that recurrence occurred within 48 months in both groups.

## 5. Conclusions

This study demonstrates that the use of Empressin during laparoscopic cystectomy for ovarian endometriomas significantly reduces the recurrence rate compared with conventional cystectomy. This effect remained evident even after adjustment for potential variables such as age, BMI, cyst size, and hormonal therapy. The combination of Empressin use and postoperative hormonal therapy resulted in a more favorable outcome, suggesting a potential synergistic effect that improves long-term disease control. However, the effect of Empressin alone was less pronounced without postoperative hormonal therapy. Further prospective randomized controlled trials with longer follow-up periods are needed to confirm these findings and explore the optimal combination of surgical and medical therapies for managing endometriomas.

## Figures and Tables

**Table 1:** Demographic characteristics and perioperative data of patients in the EIT and control groups.

Variable	EIT Group	Control Group	95% CI	*p* Value
Number of patients	(n = 110)	(n = 153)		
Age (mean ± SD)	34 ± 5.8	35 ± 6.2	−2.40–0.57	0.2
BMI (kg/m^2^) (mean ± SD)	24 ± 4.9	25 ± 5.0	−1.54–0.89	0.6
Previous surgery for endometriosis, n (%)	32 (29.1%)	50 (32.7%)	−0.15–0.08	0.5
Previous cesarean section, n (%)	10 (9.1%)	10 (6.5%)	−0.04–0.09	0.4
Preoperative hormonal therapy, n (%)	76 (69.1%)	96 (62.7%)	−0.05–0.18	0.3
Dysmenorrhea, n (%)	101 (91.8%)	129 (84.3%)	−0.002–0.152	0.1
Hypermenorrhea, n (%)	21 (19.1%)	25 (16.3%)	−0.07–0.12	0.6
Dyspareunia, n (%)	57 (51.8%)	64 (41.8%)	−0.02–0.22	0.1
Dyschezia, n (%)	42 (38.2%)	55 (35.9%)	−0.10–0.14	0.7
Dysuria, n (%)	14 (12.8%)	11 (7.2%)	−0.02–0.13	0.1
Chronic pelvic pain, n (%)	34 (30.9%)	60 (39.2%)	−0.2–0.03	0.2
Previous history of infertility, n (%)	41 (37.3%)	43 (28.1%)	−0.02–0.21	0.1
r-ASRM score				0.2
r-ASRM I n (%)	1 (0.9%)	4 (2.6%)		
r-ASRM II n (%)	14 (12.7%)	16 (10.5%)		
r-ASRM III n (%)	30 (27.3%)	59 (38.6%)		
r-ASRM IV n (%)	65 (59.1%)	74 (48.4%)		
Postoperative hormonal therapy, n (%)	63 (57.3%)	87 (56.9%)	−0.12–0.13	0.9

Notes: Data are expressed as means ± standard deviations (SDs) or numbers (percentages). CI = confidence interval; BMI = body mass index; r-ASRM = revised American Society for Reproductive Medicine classification.

**Table 2:** Results of logistic regression analysis of factors associated with recurrence risk after laparoscopic cystectomy.

Variable	OR (Odds Ratio)	95% CI	*p*-Value
Use of Empressin (yes vs. no)	0.2	0.07–0.52	0.001
Age (per year)	0.95	0.88–1.02	0.7
BMI (kg/m^2^)	0.95	0.95–1.09	0.6
O left			0.7
–Enzian O 1/0	1	0.31–3.3	0.9
–Enzian O 2/0	1.7	0.57–5	0.3
–Enzian O 3/0	1.4	0.32–5.82	0.7
O right			0.7
–Enzian O 0/1	0.7	0.21–2.5	0.6
–Enzian O 0/2	1.1	0.42–3.12	0.8
–Enzian O 0/3	1.7	0.44–6.32	0.5
Preoperative hormonal treatment	0.9	0.41–2.05	0.8
Postoperative hormonal treatment	2.4	0.96–6	0.06

Notes: OR = odds ratio; CI = confidence interval.

**Table 3:** Recurrence rate in EIT and control groups.

Time (Months)	EIT, n (%)	Control, n (%)	*p* Value
	110	153	-
6	0 (0)	6 (3.9)	0.06
12	1 (0.9)	8 (5.2)	0.2
24	4 (3.6)	18 (11.8)	0.7
36	5 (4.5)	23 (15)	0.8
48	6 (5.5)	27 (17.6)	0.8

## Data Availability

The data presented in this study are available on request from the corresponding author.

## Abbreviations

The following abbreviations are used in this manuscript:

DIE	Deep infiltrating endometriosis
EIT	Empressin Injection Technique
OMA	Endometrioma
SD	Standard deviation
